# To refer or not to refer? Exploring the cognitive process of genetic counselors' decision to refer a patient to another professional

**DOI:** 10.1002/jgc4.70223

**Published:** 2026-05-09

**Authors:** R. Beth Dugan, Kestutis Micke, Andrea Shugar, Samantha Glowacki, Beverly M. Yashar

**Affiliations:** ^1^ Department of Human Genetics University of Michigan Ann Arbor Michigan USA; ^2^ BioMarin Pharmaceutical Inc San Rafael California USA; ^3^ Division of Clinical & Metabolic Genetics and the Department of Genetic Counselling The Hospital for Sick Children Toronto Ontario Canada; ^4^ Division of Genetic, Genomic, and Metabolic Disorders Children's Hospital of Michigan Detroit Michigan USA; ^5^ Present address: BioMarin Pharmaceutical, Inc. San Rafael California USA

**Keywords:** genetic counseling, psychosocial, referral

## Abstract

Genetic Counselors (GCs) hold the dual responsibility to engage in patient‐centered psychosocial counseling and to recognize when a patient's needs require referral to another healthcare professional (HCP). While previous work has shown that GCs use a variety of patient factors to determine when they could benefit from additional supportive services, we sought to better understand how GCs determine whether to refer a patient to another HCP or engage in more complex psychosocial counseling. Utilizing a multimethods approach, we used two sets of surveys to learn about our participants but ultimately prioritized the qualitative data from in‐depth semistructured interviews with 15 participants (*N* = 15). Initial data collection and analysis relied on constructivist interpretive frameworks of grounded theory, which supported learning directly from participants while maintaining our ability to delve into issues raised by the participants. An iterative process of coding, reviewing, memoing, and testing resulted in the development of several conceptual models of possible theories. Through continued review of the data and guided by an interpretivism paradigm, we elected to honor the breadth of information shared by participants, abandoning our initial goal of defining a single theory to instead define three core conceptual categories related to GCs' consideration of providing psychosocial counseling or referring: (1) For GCs, it's personal! GC beliefs in themselves, their personal identities, and their conceptualization of the role of a “GC” influence their engagement in psychosocial assessment and support; (2) Training matters, but experience is key; and (3) Referrals occur when GCs are aware of and trust that another HCP/resource will be a good fit for a patient's need and that a patient can actually access it. These findings suggest that GC confidence in managing patient care evolves with experience and highlight the value of ongoing skill development for all practitioners.

**What is known about this topic**

Research to date has explored patient factors that influence GCs' decision to engage in psychosocial counseling or refer a patient to another HCP. These factors include limited social support, difficulty making a decision about genetic testing, a personal history of mental illness, and patient distress.

**What this paper adds to the topic**

This work assesses the cognitive processes utilized by GCs as they decide whether or not to refer patients. Findings show that GCs' clinical decision making in this regard is influenced by GC beliefs in themselves, their personal identities, their conceptualization of the role of a “GC,” and experience, as well as their trust in other HCPs/resources.

## 1 INTRODUCTION

Genetic counseling is the process of helping people understand and adapt to the medical, psychological, and familial implications of genetic contributions of disease (Resta et al., 2006). Like other regulated health professions, genetic counselors (GCs) rely on their scope of practice, practice‐based competencies (PBCs), and models of practice to guide their clinical activities and discern what interventions are within the acceptable limits of their role. A scope of practice is defined by professional organizations and used to establish laws and guide the work of regulatory bodies in determining what activities are legally allowed and should be competently performed by a member of the profession. Scope of practice can also be useful in considering how a profession functions within the network of other healthcare professionals (HCPs). For example, understanding scope of practice can help define the limits and boundaries between a GC's and a social worker's role and responsibility for a particular patient's needs.

The genetic counseling profession's guiding resources delineate dual responsibilities for GCs to provide client‐centered counseling and appropriately utilize referrals to other resources or HCPs. The National Society of Genetic Counselors (NSGC) Genetic Counselor Scope of Practice outlines that GCs provide client‐centered counseling and anticipatory guidance and identify and utilize community resources that provide medical, educational, financial, and psychosocial support and advocacy (NSGC, Institutional‐Based Model Credentialing Policy for Genetic Counselors). The Accreditation Council for Genetic Counseling (ACGC) describes seven PBCs that are considered necessary for genetic counseling, that include the ability to promote the integration of psychosocial needs and client‐centered decision making into genetic counseling interactions and to demonstrate how GCs fit within the larger healthcare system, including advocating for the continuity of care by identifying resources and/or referring to other healthcare specialties and professionals to assist a client's needs (ACGC, Practice Based Competencies for Genetic Counselors, 2023).

While these important definitions help shape the GC profession, in practice their broad descriptions provide limited guidance to GCs about fulfilling competing responsibilities. Some guidance on when to refer may come from our models of practice. One such model, the Reciprocal‐Engagement Model (REM) of genetic counseling practice defines the tenets, goals, strategies, and behaviors that guide GC practice (Veach et al., 2007). The REM offers a more granular depiction of GC practice by including specific behaviors, such as “reflect patient thoughts and feelings re: options” and strategies, such as “anticipatory guidance.” As a model, the REM helps center the counselor‐patient relationship in the GC process and outlines strategies to achieve GC outcomes. While the REM outlines important aspects of the practice of genetic counseling, as a model, it does not clarify the nuanced decisions any single counselor makes in their practice. Within each interaction with patients, GCs are continuously assessing, evaluating, and pivoting in order to understand their patient and to decide what comes next.

Research examining GC referral practices has primarily focused on patient factors that prompt a GC to refer (Cunningham et al., 2018). Cunningham's et al. (2018) work found that GCs were more likely to refer to another specialist when a patient reported a personal history of mental illness such as depression, the perception that the patient was distressed by the information within the counseling session, had difficulty making a decision, or had low social support. In considering their scope of practice, GCs described a difference in their perception of the GC and Mental Health Providers (MPH) scope of practice: namely, the GC responsibility is to provide short‐term support while MHP provided long‐term follow up. GCs acted as a filter to identify a significant issue that required referral to a MHP. From this exploratory work, additional research aimed to quantitatively assess how often referrals were made and what factors led to or acted as barriers to referrals (Hayes et al., 2022). The majority of GCs (83.1%) in this study reported that they have referred or facilitated a referral to an MHP. This study reinforced the specific scenarios in which GCs are more likely to refer, including a patient having a personal history of mental illness or having limited social support, for two examples. When considering whether or not to refer a patient to another professional, the anticipated duration (short‐term versus long‐term) was again a significant determining factor.

This work to date begins to elucidate the scenarios that trigger a GC to refer a patient to another professional. However, it is important to note the lack of universal agreement regarding referrals by study participants. While there are some situations where GCs seem to consistently support referral, such as a patient presenting with symptomatic mental illness, other situations, such as a patient having a psychological reaction to a genetic testing result, did not trigger consistent referrals (Hayes et al., 2022). Research also suggests that GCs are inconsistently utilizing formal assessment tools and that they are likely utilizing personal assessment skills including the feeling that something is “out of the ordinary” and trusting their “gut” (Cunningham et al., 2018; Hayes et al., 2022). When considering the use of patient factors and “gut feeling,” we recognize that clinician decision‐making engages both rational and emotional processes, drawing on what psychology identifies as dual process thinking (Shugar, 2020). The analytic approach involves deliberate reasoning—carefully weighing risks, benefits, and probabilities. However, many decisions are also driven by the automatic system, where instinct, emotion, and personal experience shape choices through cognitive shortcuts known as heuristics. These shortcuts can lead to clinician decisions that are made through the lens of unintentional biases that should be considered when assessing how GCs make a decision of whether or not to refer.

Given the difference in individual GC referral practices, our study sought to explore the cognitive processes GCs use to decide when to refer patients presenting with complex needs. Specifically, in the absence of an obvious reason for referral, such as mental health crisis, we investigated how GCs draw the line between providing more advanced psychosocial services versus referring to another HCP.

## 2 METHODOLOGY

We (the authors) want to acknowledge our role in reviewing, interpreting, and developing this work and the data herein, with all of our experience, expertise, and biases. We each reviewed this data independently, noting our reflections and considerations, ultimately coming together as a group to learn from one another and challenge each other's assumptions. Each of us came to this project due to a deep interest in the psychotherapeutic aspect of genetic counseling and a shared belief in its importance. As we reviewed excerpts, we identified and reflected on times when we were making our own assumptions about what a GC “should” and “shouldn't do” and considered how this was contributing to our review, occasionally challenging ourselves to let this go to truly hear from our participants. We are all board‐certified GCs, and as a group, our clinical experience ranges from 3 to 30 years, which provided us several time points across the GC experiential trajectory in providing psychosocial support. The majority of our clinical practice has been in the prenatal specialty. We include individuals practicing in Canada and the US, which provided a rich dialogue when considering how the GC practice fits within the healthcare system, available resources, and the practice of referring. Each of us is involved in GC education in some way, supervising students and/or in didactic activities or formally as program leadership, and recognize that GC training spans content from molecular genetics to counseling theories and practice. Our interest in this project and the lens through which we defined what was important to share in this manuscript are impacted by our desire to improve experiential learning in how to create patient‐centered, psychosocially supportive environments.

This work occurred in two phases: Phase 1. The initial study was designed and data collected as part of a Master's of Science student's (Braeden Hughes) thesis work at the University of Michigan Genetic Counseling Program, and Phase 2. as a continuation by the listed authors (we) who subscribed to an interpretivism paradigm in returning to and reflecting on the data that had been collected. We sought to understand the GC experience in engaging with psychosocial counseling and considering referral, prioritizing their experience. The study used a multimethods approach, utilizing both quantitative and qualitative data to learn about the experience of GCs contemplating referral. In review of the data and the definition of conceptual categories, we relied on a constructivist framework of grounded theory, which helped guide our consideration of the varied experiences of participants in conjunction with reflection of our individual and group perspectives.

### 2.1 Participants

In order to focus specifically on cognitive processes and minimize additional variables, such as specialty, we focused on recruiting GCs from prenatal practice. Certified GCs who currently or previously practiced in prenatal genetic counseling were eligible for this study. Study participants were recruited through the NSGC Research Survey E‐Blast and through email contacts provided by the authors' professional networks. According to the 2022 NSGC PSS survey, 636 respondents worked in direct patient care or mixed positions in the prenatal specialty. Eighteen (*N* = 18) individuals responded to the initial survey and received an invitation to interview.

### 2.2 Instruments

A “screener survey” was created to collect demographic information from potential participants. This survey collected contact information, years of experience, and asked participants the following questions: “How comfortable are you using psychosocial skills during a genetic counseling session?” The original intent of the “screener survey” was to select participants who had a range of years of experience and comfort with psychosocial counseling to interview. Ultimately, all participants who completed the “screener survey” were invited to interview. The “screener survey” was distributed using QualtricsXMA on November 30, 2022, with a reminder on December 7, 2022.

A second survey, a “scenario survey” was created to ground interview participants with a common point of reference that explored the GCs' cognitive process when faced with different factors that may arise in a session. This survey provided participants with six different scenarios. For each scenario, participants were asked whether they would refer the patient to outside services, would not refer the patient to outside services, or were uncertain about referring. Participants were also asked to rate their decisional confidence on a Likert scale (1 = not at all confident, 10 = very confident). The scenarios were built around a core case description and then modified in one key variable (Supplemental Materials). Variables included patient concerns related to: financial crisis, partner discordance, cultural concerns, patient intellectual disability, patients' mental health diagnosis, and spiritual/religious crisis. The “scenario survey” was piloted by three GC students familiar with prenatal counseling. No changes were made following the “scenario survey” pilot. The scenario survey was provided to participants prior to the interview. The scenario survey was administered using QualtricsSM.

Constructivist grounded theory guided the design of the semistructured interviews. This method supported learning directly from participants and remaining flexible in the ability to follow up on perspectives shared in a conversational, natural style. The interviewer (BH) utilized the case scenarios as a focus of shared meaning but did not remain fixed to or rigid in their questions for participants; instead, questions remained broad, focusing on the participants' experiences and thoughts as they considered the scenarios. The content of the interview guide was informed by existing literature on GCs' referral decisions and committee members' experience with referral in the prenatal setting. The interview guide included questions exploring the participant's thoughts when they considered possible referral versus the provision of support in each scenario, including asking which scenarios provided the most difficulty when deciding when to refer.

### 2.3 Procedures

Semistructured interviews were conducted between December 2022 and January 2023, generating qualitative data through inductive and deductive approaches (Corbin & Strauss, 2008). During the interview, participants answered broad questions about their education and their experience and comfort with psychosocial assessment and support. Following initial interviews, the interviewer continued to tailor their approach to dive deeply into the areas of interest for this project, which became more focused on key aspects of education and GC‐held ideas of scope. The multimethods approach provided participants with scenarios to reflect on prior to the interview. This allowed the interviewer to move quickly into deeper questions, eliciting richer responses from participants. Interviews lasted between 35 and 64 min and provided direct insight into the research questions. The tailoring of questions and the ability to move quickly from a shared foundational starting point allowed for a high degree of informational power, in which each interaction provided high‐quality, rich data that provided ample insight into our research objectives (Malterud et al., 2016).

### 2.4 Data analysis

Interviews were conducted and transcribed over Zoom (version 5.12). Transcripts were edited for accuracy using Microsoft Office transcription services and uploaded to Dedoose (Version 9.0.17) qualitative data analysis software (SocioCultural Research Consultants LLC; California, USA). Inductive and deductive coding were applied to review and process the data. Prior to coding, potential codes were defined based on review of available literature regarding genetic counseling practice in referral and psychosocial practice. Codes were redefined and edited following initial review of the first three transcripts by three committee members (master's student Braeden Hughes, authors BMY and RBD). Codes included Patient Factors, Genetic Counselor Factors, Emotions, Session Factors, Referral Factors, and Clinical Examples. Transcripts were reviewed independently by at least two committee members (master's student Braeden Hughes, authors BMY and/or RBD), and code consensus was reached through discussion.

The authors (we) reviewed all coded transcripts. We began by each reviewing excerpts within a single code, making memos in a shared file regarding our perceptions of the participants' meanings and reflecting on our own experience, either as a clinician and/or educator. We then met to discuss and deliberate the meaning and significance of participants' responses. Through this process of discussion and deliberation, we summarized key points from the participants' responses within a single code. Once several individual codes were reviewed, we reviewed key take‐aways across codes, first individually noting our impressions of the data in a shared file and then meeting to discuss. The goal of the group meetings was not consensus but to deepen our own consideration of the data and to develop conceptual categories. While the initial scenario cases included broader circumstances that might prompt different types of referrals, we made the decision in analysis to focus on categories that illustrate the cognitive process of the GC as they consider providing additional support themselves or making a referral to an outside HCP or resource. This decision was made based on gaps in the existing literature and our interest in this area. As part of the analysis, several conceptual models of potential theories were developed. With each model, we revisited individual codes to consider whether the model appropriately incorporated the categories we wanted to define from our participants' experiences. Through several iterations, we made the decision to honor the quality and breadth of the data we had reviewed and opted in this manuscript to define three core conceptual categories (CCCs) rather than a single theory. These CCCs could be utilized in future works with the goal of the creation of a theory.

## 3 RESULTS

### 3.1 Participant demographics

Eighteen GCs completed the initial screener survey. All 18 reported being at least somewhat comfortable with psychosocial counseling (Table 1). Additional participant characteristics were variable including location of practice, healthcare setting, and the types of indications seen in the clinic. All 18 were invited to participate in an interview and completed the scenario survey, while 15 individuals agreed to participate in interviews. Interviewed participants ranged in experience from <1 year to over 15 years of experience.

**TABLE 1:** Interview participant demographics.

Characteristics	*n* (%)
Years of experience	
<5 years	8 (53%)
5–14 years	3 (20%)
15+ years	4 (27%)
Comfort with psychosocial counseling^a^	
Extremely comfortable	5 (33%)
Somewhat comfortable	10 (66%)
Neutral	0 (0%)
Somewhat uncomfortable	0 (0%)
Extremely uncomfortable	0 (0%)
Location of practice	
United States	7 (47%)
Canada	8 (53%)
Healthcare setting	
Specialty center	4 (26%)
Public/community/safety‐net hospital	4 (26%)
Outpatient clinic/MFM office	3 (20%)
Private hospital	1 (7%)
Did not state	3 (20%)
Types of indications seen	
Mostly high risk	7 (47%)
Mixed risk	3 (20%)
Mostly low risk	2 (13%)
Did not state	3 (20%)

^a^ Results from Screener Survey.

### 3.2 Referral practices

The majority of respondents to the scenario survey (*n* = 18) would refer to additional services in each scenario (Appendix S1, Table S1). Examples of referrals included psychiatry, social work, community health workers, Cleft Lip and Palate Program, spiritual care/chaplain services, couples'/family counseling, and others.

Considering the scenario variables (Appendix S1, Table S1), participants were most likely to refer in cases of Psychiatric Diagnosis (*n* = 14), Spiritual/Religious concerns (*n* = 13), and Financial Concerns (*n* = 12). Fewer participants, though still the majority, would refer in the scenarios involving Cultural Concerns (*n* = 10), Intellectual Disability for the patient (*n* = 9), and Partner Discordance (*n* = 8).

During the interviews, participants (*n* = 15) elaborated upon the factors that influenced their decision to refer. These included mental health concerns, financial concerns, and when additional referral was requested by the patient. Many participants described these as “red flags,” prompting a referral with high confidence. Another “red flag” included the patient seeming “stuck,” which was described as the patient not progressing toward a decision or adaptation to a decision or result.

Participants described situations that fall within the GC skillset to manage, including patients stating that they have an adequate support system, that the patient response to information/diagnosis seems “appropriate,” and that the patient is showing progress in decision‐making or coping.

Patient factors in which participants were more divided in their decision whether or not to engage in more advanced psychosocial counseling or to refer to another HCP included the patient holding specific religious backgrounds that influence care, the patient having a history of intellectual disability impacting decision‐making abilities, decisional conflict between patient and support persons involved, and when patient verbal responses and action are inconsistent.

### 3.3 Cognitive processing for placing a referral

We defined three CCCs that illustrate the cognitive process GCs undergo when contemplating engaging in psychosocial counseling or referring to another HCP: (1) For GCs, it's personal! GC beliefs in themselves, their personal identities, and their conceptualization of the role of a “GC” influence their engagement in psychosocial assessment and support, (2) Training matters, but experience is key, and (3) Referrals occur when GCs are aware of and trust that another HCP/resource will be a good fit for a patient's need… and that a patient can actually access it. Table 2 provides representative examples that support each CCC.

**TABLE 2:** Genetic counselor cognitive process in considering referrals.

Core conceptual categories (CCC)	Examples
For GCs, it's personal! GC beliefs in themselves, their personal identities, and their own conceptualization of the role of a “GC” influence their engagement in psychosocial assessment and support	GCs have an individual, innately‐held belief that they “are” or “are not” a psychosocial provider. This belief is foundational to how much a GC engages with psychosocial training and psychosocial issues in a session
	GC relies on personal identities and experiences (religious, personal mental health, culture, financial resources, etc.) to engage with patients. When a perceived patient need results from identities or beliefs that are discordant with the GC's, GCs are less comfortable providing support
	GCs conceptualize their personal interpretation of scope and the balance between the educational and psychosocial roles
Training matters, but experience is key	GCs' conceptualization of their role in psychosocial support is initially informed by their graduate training and education, which is driven by the ACGC competencies
	Confidence in psychosocial engagement is built from successful and unsuccessful patient encounters. GCs will integrate skills that worked in successful encounters and avoid skills that did not work in unsuccessful encounters
	Interactions with colleagues support a GCs understanding of scope with respect to psychosocial counseling
Referrals occur when GCs are aware of and trust that another HCP/resource will be a good fit for a patient's need, and that a patient can actually access it	GC is able to identify a resource (psychotherapist, financial counseling, chaplain services, etc.) and believes that resource will better meet the patient's needs
	GC considers their responsibility in placing a referral and ability to make the referral as compared to another HCP being responsible for placing a referral
	GC weighs the burden that a potential referral may place on a patient (financial, time commitment)

#### 3.3.1 CCC1: For GCs, it's personal! GC beliefs in themselves, their personal identities, and their conceptualization of the role of a “GC” influence their engagement in psychosocial assessment and support

GCs have innate characteristics or personal identities that influence their decision to refer or engage in deeper psychosocial support. When considering their practice, some GCs shared that they felt they had a natural or innate inclination for more complex psychosocial support of patients. Other GCs reported the opposite, feeling innately not inclined to engage with complex psychosocial support. This influenced elements of their training, such as picking an educational program that seemed to emphasize (or had less emphasis on) psychosocial elements of genetic counseling or their engagement with complex psychosocial content. Participants shared that this underlying innate characteristic compelled them, some toward psychosocial engagement and some away from providing this content:

First and foremost, I'm a counselor, so that's what drew me to this field. That's why I wanted to do this. I always loved science. I always loved genetics. But I wanted to be managing the emotional needs of my families, not the medical needs of my patients. So that's really just what drew me to the field anyway in general, so I think that that probably is just the way… that's the way I do life. That's the way I manage my friendships, my personal relationships. I think that's my view on life, so I guess that's just my approach.(Participant 18)

I've always really struggled with that part of genetic counseling. That's definitely my weakest point, starting from grad school. That was where I needed improvement the most, (laughs) so I feel like it's good to obviously, always try to grow professionally as a provider, but also like accepting who I am as a counselor and what I do bring to patients and what I don't bring to patients. (Participant 6)

Participants also described relying on their identities and personal experiences to connect with a patient and engage in more complex psychosocial support. For example, if a GC and a patient share a similar religious identity, the GC may feel more comfortable engaging in further discussion and support. Conversely, if the GC felt that their patient had a different religious background, they reported feeling less capable of supporting the patient and more likely to refer to someone more familiar with this identity. This includes various personal identities.

Oh yeah, the one with the cultural thing with the patient from a different culture than my own who is worried about how her family and support system would react… I think, yeah, that would be difficult for me. That's something that I don't have a ton of experience with. I think a lot of patients would not share something like that with me, especially when an interpreter is used. I would say I… probably am missing out on some of those feelings that patients might have, and if they did share it, I wouldn't really know how to respond well, in a way that would be helpful to them. So, I would try to find a provider from a similar background. I work in a large group, so I would be a little bit concerned about what I'd be able to find someone, even in my large group from that same background, and would they be receptive to talking to my patient? But that was my thought process there. (Participant 6)

I'd say religion sometimes… makes me a little… because I'm not religious, so I don't really have a lot to relate to. And I don't know the intricacies of every religion, and I don't know if the patient is telling me the truth. They might just be saying it because they want to do what they want to do. (participant 7)

When considering their practice and the decision to refer, participants discussed what they saw as the role of the GC. Participants described their concept of scope and the need to balance educational and psychosocial factors in their sessions. Many participants described the importance of education within a genetic counseling session as this provides a foundation for patients to understand and facilitate decision‐making. However, the majority of participants reflected the belief that GCs also have an obligation for basic psychosocial assessment and support. This seems to reflect more what a participant thought GCs in general should do, but not always what they were personally comfortable doing in practice. Ultimately GCs differed in their personal determination of what level of educational versus psychosocial support is appropriate for a given scenario.

I see my role as a GC to explain test results or ultrasound findings to individuals and help them, in a nondirective manner, come to and decide on next steps. And hopefully do that in a way that feels like a… safe space and make them feel like this is really a decision that they are making. And I've always felt that we're dealing with really difficult scenarios. They're going to encounter this anyway, so how can I alleviate some discomfort, some stress, some bad feelings. That that's my role. (Participant 8)

I think my role is to give information, help people understand their options, empower them to make their own decisions. I think part of our role is advocating for patients' decisions, when other members of the team don't understand why a patient made an informed choice to not do testing or to do testing and might be questioning that. I think a lot of what we do is advocating for patients and why they've made that decision. And in terms of psychosocial, I would say our role is to definitely provide some support, maybe more as it relates to the question at hand about testing or diagnosis or something. But that our scope kind of ends when it gets outside of the realm of where we were trained in terms of providing psychosocial support, which is probably different for everybody. (Participant 14)

#### 3.3.2 CCC2: Training matters, but experience is key

GCs utilize experiences from both training and professional activities to evaluate their ability to meet the psychosocial needs of the patient. Participants shared that they reflect on content and experiences gained in graduate training as well as failures and successes on the job to inform their decision of whether to engage in more complex support or refer a patient to another HCP. Participants shared that their idea of psychosocial support in the context of genetic counseling was first informed by their graduate training which is driven by competencies defined by the ACGC. This is reflected in responses when asked what they thought about when considering referral in the provided scenarios:

I guess in general, I was thinking a lot about the training that I received “…” in my graduate training. You know, what kind of things did I learn, what are my qualifications as a GC? But then, where is the most appropriate line to draw for kind of referring patients elsewhere? (Participant 3)

We had a pretty heavy focus on psychosocial in our program, and we had the specific classes where we focused on different types of counseling theories and practice quite a bit on how to help people make decisions, using motivational interviewing was one of them. Do I use it every day? No, but in the cases where it's difficult, I kind of go back to think about OK, what was I taught in this course in this class, and how can I use that now? So that's what I thought about when I was picking between the scenarios. Was it something that I have been prepared for, or is something that's completely new, out of my scope, outside of the counseling aspect of it, then that's when I decided to refer to somebody else. (Participant 10)

Interviewees discussed job experience, including successful and unsuccessful patient encounters, as an important factor for building their confidence in providing psychosocial support and in defining the bounds of psychosocial support in genetic counseling. Participants commented that their education built a foundation in their understanding of psychosocial assessment in genetic counseling but that the ability to provide this more nuanced, complex service grew with experience. Participants also shared that practice allowed them to recognize and accept the limits of their psychosocial support:

Yeah, I mean sometimes I definitely feel out of my depth in terms of, you know, how best to help. Most of it is just experience, right? Sometimes (I) have students rotating with me and they'll come and like “oh. How do you know what to say? You're so good at this!” Yeah! That's because I've had 16 years of practice doing this exact thing, you learn. The knowledge, sure, but the themes that come up, the things that seem to work and don't work with different patients. I mean, it's just building a repertoire, my toolbox continues to grow. You just have to figure out what works, what doesn't, what works sometimes… also always self‐assessment. “Why did it work this time with this patient but it didn't work with the other patient.” I do now feel like… not that I can tackle everything, but I can certainly tackle a lot more now than I could at the beginning of my career. (Participant 18)

I think that when I was fresh out of school, I spent a lot more time and really tried to delve into things deeper with patients. And I think now I have a more healthy boundary of what I'm able to do and not do. And really, I think that… I have a different impression of where I draw the line. In general, when you're starting out of school, you have this go‐getter attitude and you're bright‐eyed and bushy‐tailed, and you want to apply everything that you've learned and everything is a psychosocial experience, and then you realize that you can't do that and get home on time and make dinner. And sustain yourself. (Participant 8)

In addition to education and experience, participants report that their understanding of the GC's scope of practice in psychosocial counseling was shaped and affirmed by their interactions with their colleagues:

Talking it over with coworkers I think can be helpful. Even when I'm unsure, and I'm like, “I'm seeing these green or red flags” and I'm like debating, I'm like, “oh gosh.” I think something really helpful that I've done is talk to my coworkers. I have a prenatal coworker who's been at our Center for like 15 years. That's something that's really helpful to me. Or if I go and talk to the fetal care center, I talked to our nurse coordinator and usually they also pick up on the signs… it's that gut feeling that I talked about that is not specific. And unhelpful but… I feel like It's just continually being developed. (Participant 1)

#### 3.3.3 CCC3: Referrals occur when GCs are aware of and trust that another HCP/resource will be a good fit for a patient's need… and that a patient can actually access it

GCs' referral practice is influenced by their awareness of and confidence in a resource's ability to meet a patient need, their ability to place a referral, and potential barriers to access for the patient. Once GCs recognize an emerging issue in a session, they consider their ability to identify another HCP whom they believe will be able to better meet the unique patient need. This includes identifying the type of professional whose scope of practice might best address this need (psychotherapist, financial counseling, chaplain services). Beyond identifying the type of professional, participants described considering whether or not the service is better equipped (than themselves) to handle the need. The perception of the service being “better” is informed by their own personal experience and feedback from patients:

We have a really great pastoral care team at the hospital that I work at, and they are often involved in patients of ours who have terminations by… induction of labor. And patients who are religious or patients who aren't religious but want some sort of spiritual blessing of the baby, the pastoral care is often involved, so it's a pastoral care team that they have this religious background, but they're very supportive in terms of how nuanced these decisions are and how challenging they can be, especially for patients who do have these really strong religious beliefs. And so, I think that's why I felt more confident is because I know what they would get on the other end of it. I think whereas that lack of confidence with that other patient was more… I don't know what I would be. I would be referring into a void, and I don't know what they would be getting. I think some of the confidence comes in knowing who you're referring to and knowing the type of care that they would receive.(Participant 15)

So, coming hot out of grad school… you know, the first couple of years, I definitely felt like… I had more ego involved… and I think it's natural for a lot of newer GCs to feel like, “well, if I don't make this happen, who is going to?” You know? And me now, it's like look, I know my scope and I'm going to stay squarely in it. You know… there's other people who are better situated for certain areas of care, and I need to trust that they are also going to be competent at their job. You have to build trust in the system, and you know there's holes, but you can't fix those holes systemically. You can't fix those holes by burning yourself out. And so, you need to give more of that responsibility to the other people in the healthcare team. (Participant 4)

When considering referral, participants also shared differing perceptions of their role in placing referrals and their ability to do so. Some participants shared that GCs in their practice do not place referrals. Other participants shared that they make referrals and that community and/or institutional systems in place aided in their ability to make a referral. Finally, some GCs expressed their belief that a patient's primary care physician should be the one to place a referral as they have a more established relationship with the patient:

Mostly, in my position in my clinic, I have never referred a patient anywhere else. That's all up to the provider managing the pregnancy, whether it's one of the OB docs, or the MFM or the midwife. I think we have a couple of PAs, although mostly midwives. It's other decisions… I might go talk to the MFM or the midwife, in fact. I'd be fairly likely to do that, but I myself would not place any referrals. (Participant 16)

Right, and part of my confidence in being able to refer them is that I'm fortunate to work in a very large tertiary care center where we have some of the support built in institutionally, available at the institution. I'd probably have a much harder time if I was say working in sort of a freestanding clinic or you know in a much smaller practice, but I'm fortunate that you know we have a dedicated social worker to our clinic. We have a hospital chaplain. We actually also have just on the floor below where my clinic is, we have a dedicated reproductive psychiatry clinic. The… so… I feel confident in referring because I physically know that I have all of those resources directly available to me versus having to keep more of a read on… out in the community and kind of pull together resources piece meal. (Participant 4)

Prior to placing a referral, participants considered the burden that the potential referral may place on the patient. This included consideration of the financial burden of the service as well as the time commitment required of the patient. Concern for these burdens made some participants less likely to refer to another HCP or made them consider which resource was most accessible for their patient:

I think there's always some challenges with referrals. I think… I mean at that point the patient would be needing to make another appointment. Sometimes if they don't have a lot of availability in their schedule, maybe they're less likely to be able to do that. All those accessibility issues with, you know, taking time off of work, transportation, all that kind of stuff might not be feasible for a lot of people. So, that's certainly another reason, and I think when you get more people involved, it just sometimes gets a little more complicated with, you know, sending appropriate records to the right place and then making sure that place is contacting the patient. Or is it the patient that needs to contact them? Sometimes things can certainly get lost. I've definitely witnessed that in the past. (Participant 3)

I refer to this person. Well, it turns out that person costs $350.00 an hour to provide therapy and counseling for loss so, you know. And she doesn't take insurance, so that's accessible to some of our patients, but not too many of them. So, I think because of all of that I've been referring people more to support groups rather than individual providers, because I know that then insurance isn't really an issue. It's a lot more affordable. There [are] many free options, and it's a lot easier to find a support group that's really tailored to a specific thing than it is to find a mental health provider that's really focusing on a specific thing and that that works with someone's insurance. (Participant 6)

## 4 DISCUSSION

When considering providing complex psychosocial assessment and support, GCs participating in our research shared several patient factors that made them more or less likely to refer. These included factors that made them less likely to refer, such as the patient verbalizing having an adequate support system, and factors for which they were most likely to refer, such as the patient having significant mental health concerns or difficulty making a decision. These factors are concordant with those identified in earlier research (Cunningham et al., 2018). The analysis presented in this work focused on understanding the GC cognitive process in nuanced situations where the GC contemplated engagement that utilized more complex psychosocial support or referring to another HCP. When considering complex psychosocial counseling, GCs were influenced by what they thought a GC should do, in theory, as well as what they personally were comfortable doing in practice. Participants described a belief that some level of psychosocial assessment and subsequent support is part of their responsibility but held different opinions regarding the appropriate level and the extent of support they were comfortable personally providing in their sessions. Within their personal practice, GCs weighed how psychosocial support balances with the educational demands of their role within their understanding of their scope overall. The GC field has long discussed the competing demands of our roles in education and psychosocial support. Early models seem to put the teaching and counseling roles of the GC at odds with one another while later models, such as the REM, seek to embed the two concepts into a single paradigm that complement each other: teaching is informed by an empathetic, person‐centered approach that, in turn, supports psychosocial adaptation (Betzler & Roberts, 2025; Veach et al., 2007). While this approach may be instructive in more straightforward GC sessions, for some situations, where the patient is struggling to meet the GC outcomes described by the REM (i.e., patient decision making and adaptation), GCs may reach the end of their ability to offer further psychotherapeutic care. Some participants described the importance of experience in gaining an appreciation of the outer boundaries of their ability. As a novice GC, some desired doing it all for patients but, with time, they began to define their limits, either due to external factors, such as patient load and time, or internal factors, such as increasing confidence in their ability to provide psychosocial counseling. Recognizing these boundaries is essential in ensuring patients receive appropriate care. There is risk, however, that defining boundaries too narrowly ground GCs in a teaching‐only model or that factors other than patient needs, such as time, dictate a GC decision regarding the fulfillment of their psychosocial roles. Patients express higher satisfaction when clinical practice emphasizes counseling over teaching (Austin et al., 2014). For this reason, GCs should take care when considering their psychotherapeutic boundaries.

The decision to refer is not only based on the participant's belief that they can/cannot meet the identified need, but the knowledge and belief that there is another HCP who is better suited to meet this need. GCs in this work saw themselves positioned in the healthcare system where there are boundaries to the roles they perform and those of other HCPs and resources. Referral requires the GC to know of other professions and their scope of practice. To best prepare them to enter the profession, educational programs have a role in introducing students to other healthcare professions and their roles, such as Social Work and Chaplain Services/Pastoral Care. This requirement can be seen in ACGC's Practice Based Competencies in Domain 6: Demonstrate how GCs fit within the larger healthcare system (Accreditation Council for Genetic Counseling Revised Practice‐Based Competencies, 2023). This study suggests, however, that knowing of another profession (in general) is not enough for GCs to make a referral, even when they question if they can meet a specific patient need. Participants described wanting to have some experience with the HCP/resource they would be referring to and expressed a sense of increased comfort with referrals when they are familiar with a referral process. Following graduation and when entering a new clinical space at any point in their career, GCs may benefit from becoming familiar with their community, both within their healthcare system and outside of the system, such as community resources. This could begin with an exploration of available resources within one's healthcare system but may go further by reaching out to specific individuals to understand what a referral or collaboration might look like.

Identifying specific HCPs and/or resources within one's own community may be especially important when addressing religious or cultural concerns. In this work, GCs contemplating providing services or referring considered whether the client need is related to an identity or personal experience shared by the GC, which might tip the scale in favor of the GC feeling they can meet this particular need. The majority of participants (86.7%) indicated that they would refer to another HCP when patients have a religious/spiritual concern. Previous work suggests that GCs do not use formal assessment tools and that they are likely utilizing personal assessment skills and trusting their “gut” (Cunningham et al., 2018; Hayes et al., 2022). As a profession that is predominantly heterosexual (86%) cis (99%) white (87%) women (92%) who identify as agnostics (24%), nonreligious (18%) or atheist (15%) (NSGC, PSS, 2024), practitioners should question what biases they hold when deeming something as “out of the ordinary,” such as deciding that a patient's grief is “abnormal.” Engagement with community‐based learning can lead to increased confidence in providing care to patients with identities differing from our own (Ernst et al., 2023). This is a space where training programs as well as individuals can expand their awareness of cultural impacts on behaviors, such as medical decision making and grief, to aid in appropriately and effectively recognizing the need for additional support and potential referral to another HCP. Establishing ties with individuals within the healthcare system and community may increase GCs utilization of other potential resources and their ability to identify the best fit for their patients' needs. Furthermore, efforts to increase diversity in the genetic counseling workforce would produce GCs who hold a variety of identities that might better support the psychosocial needs of all our patients.

Participants describe an evolving definition and growth in their ability to provide psychosocial counseling with increased experience. Graduate training programs had a role in setting the foundational understanding of what constitutes counseling within the GCs' scope of practice; but participants described adjusting the conceptualization of this role with practice and in interactions with colleagues. GCs described a trial‐and‐error approach to psychosocial assessment and support, where they learned from their successes and failures in their approach to counseling in sessions. This suggests that, while educational programs have a role in providing content on counseling theories and an opportunity to practice in clinic, psychosocial skills are likely to continue to develop over the course of one's career. For this reason, psychosocial assessment and support may require continued development through practice and intentionality after graduation. Furthermore, without specific attention to this skillset, GCs may default to an education model out of comfort or ease, neglecting a potential area for professional growth and patient satisfaction. Advanced psychosocial counseling skills may be an ideal space for career‐long, potentially peer‐to‐peer, supervision. As outlined by a recent publication by McEwen et al. (2025), career‐long supervision is a professional activity that includes a process of review, reflection, learning, critique and replenishment for practitioners. Supervision in psychotherapy has been associated with skill acquisition and utilization by practitioners (Wheeler & Richards, 2007). This reflective practice may be especially beneficial for specific skill development and practice throughout one's career, such as advanced psychosocial assessment and support. Furthermore, requirements for career‐long supervision may legitimize and protect time for dedicated attention to these skills. With the prolonged development of advanced psychosocial skills extending beyond graduation, the profession might benefit from the development of a variety of educational resources, practice tools, and methods for evaluating continued competence in psychosocial counseling skills. Of note, we did not attempt to define “experience” for participants. Future studies could explore the impact of years of experience and of different types of education or development activities on GCs' psychotherapeutic practice and referral patterns at different points across the professional trajectory to make further recommendations.

While our original intent was to develop a theory that would describe this cognitive process, we abandoned that objective for several reasons. We included a convenience sampling with interviews of 18 GCs. We believe the perspectives shared have highlighted important concepts that should be explored in further research that might contribute to the development of a theory. We did not explore the identities of participants in depth, identities that might influence their psychosocial practice. All participants were from North America; psychosocial practices of GCs in other countries and societies may not be represented in this work. Our participants reflected on their practice in the prenatal setting and we did not specify service delivery models. Specialty‐specific psychosocial considerations may also have influenced the categories identified. Future studies could explore these CCCs in other specialties with exploration of the impact of service delivery models prior to the development of a theory, ensuring broader representation of the GC process.

## 5 CONCLUSION

Ultimately, our study reaffirmed previous work which demonstrated knowledge of patient concerns which GCs recognize are beyond their skillset and scope of practice to manage. Also supporting prior studies was our finding that there are numerous situations which do not fall into a clear binary refer or do not refer decision. In these situations, GCs are forced to personally determine if they have the skills to manage the concern, or if it falls outside their abilities and requires the involvement of another professional. As a result of our qualitative data gathered through semistructured interviews, we identified factors that GCs use when processing the decision to keep or refer a patient. Broadly, we found that GCs rely on innate and personal characteristics, formal and informal training, and knowledge of whom they are referring to in making this decision. Our data also suggest that GC confidence in placing a referral or managing a patient evolves with experience. Thus, a consideration for continued skill development in this part of our practice may include integration of scenario‐based practice and/or peer supervision to ensure GCs are operating both within and at the top of our scope of practice.

## AUTHOR CONTRIBUTIONS

We worked collaboratively in interpretation of the study's data with key contributions from each author. R. Beth Dugan and Kestutis Micke drafted several conceptual models for group processing of the data. R. Beth Dugan drafted the manuscript and has full access to the data in the study, taking responsibility for the integrity of the data and accuracy of the data analysis. R. Beth Dugan, Beverly M. Yashar, Andrea Shugar, Samantha Glowacki, and Kestutis Micke were involved in study design and made significant contributions in critically assessing the data, analysis, and revising the manuscript. All authors gave final approval of this version to be published and agreed to be accountable for all aspects of the work.

## FUNDING INFORMATION

Funding for this work was provided by the University of Michigan Rackham Graduate Student Research Grant.

## CONFLICT OF INTEREST STATEMENT

Authors R. Beth Dugan, Kestutis Micke, Andrea Shugar, Samantha Glowacki, and Beverly Yashar declare that they have no conflict of interest.

## ETHICS STATEMENT

Human studies and informed consent: Human studies and informed consent: This study was reviewed and granted exemption by the Institutional Review Board of the University of Michigan Medical School (IRBMED) (HUM00223717). All procedures followed were in accordance with the ethics standards of the committee responsible for human experimentation (institutional and national) and with the Helsinki Declaration of 1975, as revised in 2000. Informed consent was obtained from individuals who completed the online survey and who participated in interviews.

Animal studies: No nonhuman animal studies were carried out by the authors for this study.

## Supporting information


Appendix S1

## Data Availability

The data that support the findings of this study are available from the corresponding author upon reasonable request.
