# Supplementary material for: To refer or not to refer? Exploring the cognitive process of genetic counselors' decision to refer a patient to another professional
Source: J Genet Couns. 2026 May 9;35:e70223. doi: 10.1002/jgc4.70223 (PMC13157526; doi:10.1002/jgc4.70223)
Supplement: Supplementary file 1 — Appendix S1 [file JGC4-35-0-s001.docx]

**Supplemental Materials**

**Scenario Survey**

Core Scenario

You meet with a patient to discuss the findings of her 20-week anatomy scan. The sonographer noted a unilateral cleft lip during the ultrasound. There is no family history of similar concerns. You discuss with the patient the likelihood of syndromic vs. non-syndromic cleft lip. She expresses that she is extremely anxious about this finding and the potential for the fetus to have a genetic disorder.

| Variable | Description | Refer Out (% of respondents who would refer to another HCP |
| --- | --- | --- |
| Psychiatric Diagnosis | She shares that she has a diagnosis of bipolar disorder type I and that managing her mental health on top of processing this finding has been challenging. She explains that she is terrified of something happening to the fetus and that at times she feels out of control. | 93.3 |
| Religious/  Spiritual Concern | When you discuss the option of termination with her, she discloses that she does want to terminate but that she knows that she would be ostracized by her religious community if she did so. She begins to cry and states that she feels like she has been abandoned by God and that she is going to Hell. | 86.7 |
| Financial Concerns | She shares that she doesn’t have health insurance and that she is struggling to pay her bills each month. She states that having a child with significant medical needs would be financially devastating, but she does not want to terminate or consider adoption. | 85.7 |
| Cultural Considerations | She states that in the country where she is from, children with disabilities are looked down on and frequently mistreated. She feels that her family and her culture would never accept a child with these differences. She is concerned about losing her support system and her identity if she continues with this pregnancy but doesn’t want to terminate or explore adoption. The session was conducted with the aid of an interpreter. | 62.5 |
| Intellectual Disability | She has an intellectual disability and is struggling to understand all the information doctors have already thrown at her. She states that she feels overwhelmed and confused by everything and doesn’t know what to do | 60.0 |
| Couple Discordance | When you discuss the option of termination with them, your patient discloses that she does not want to terminate. However, her partner feels that it would be unfair to their other children to have a child with significant medical needs and would prefer to end the pregnancy. The conversation is becoming emotional, and despite your efforts to support them, the couple seems unable to come to a resolution together. | 53.3 |

**Table 3. Scenario Variables in order of percent of respondents who would refer to another HCP (highest to lowest)**
